# Attogram-level light-induced antigen-antibody binding confined in microflow

**DOI:** 10.1038/s42003-022-03946-0

**Published:** 2022-10-06

**Authors:** Takuya Iida, Shota Hamatani, Yumiko Takagi, Kana Fujiwara, Mamoru Tamura, Shiho Tokonami

**Affiliations:** 1 Department of Physical Science, Graduate School of Science, Osaka Prefecture University, 1-2 Gakuencho, Nakaku, Sakai, Osaka, 599-8570 Japan; 2 Research Institute for Light-induced Acceleration System (RILACS), Osaka Prefecture University, 1-2 Gakuencho, Nakaku, Sakai, Osaka, 599-8570 Japan; 3 Department of Applied Chemistry, Graduate School of Engineering, Osaka Prefecture University, 1-2 Gakuencho, Nakaku, Sakai, Osaka, 599-8570 Japan; 4 Graduate School of Engineering Science, Osaka University, 1-3 Machikaneyama-cho, Toyonaka, Osaka, 560-8531 Japan; 5 Present Address: Department of Physics, Graduate School of Science, Osaka Metropolitan University, 1-2 Gakuencho, Nakaku, Sakai, Osaka, 599-8570 Japan; 6 Present Address: Research Institute for Light-induced Acceleration System (RILACS), Osaka Metropolitan University, 1-2 Gakuencho, Nakaku, Sakai, Osaka, 599-8570 Japan; 7 Present Address: Department of Applied Chemistry, Graduate School of Engineering, Osaka Metropolitan University, 1-2 Gakuencho, Nakaku, Sakai, Osaka, 599-8570 Japan

**Keywords:** Nanoscale biophysics, Biochemical assays

## Abstract

The analysis of trace amounts of proteins based on immunoassays and other methods is essential for the early diagnosis of various diseases such as cancer, dementia, and microbial infections. Here, we propose a light-induced acceleration of antigen-antibody reaction of attogram-level proteins at the solid-liquid interface by tuning the laser irradiation area comparable to the microscale confinement geometry for enhancing the collisional probability of target molecules and probe particles with optical force and fluidic pressure. This principle was applied to achieve a 10^2^-fold higher sensitivity and ultrafast specific detection in comparison with conventional protein detection methods (a few hours) by omitting any pretreatment procedures; 47–750 ag of target proteins were detected in 300 nL of sample after 3 minutes of laser irradiation. Our findings can promote the development of proteomics and innovative platforms for high-throughput bio-analyses under the control of a variety of biochemical reactions.

## Abstract (web summary)

Attogram-level of proteins can be detected using light-induced acceleration of antigen-antibody interactions in a microchannel platform.

## Introduction

Proteins, initially discovered in 1838, are crucial nano-biomaterials involved in various vital activities^1,2^. Their structural analysis has greatly furthered the progress of the biomedical field^3,4^, and new research is daily updated in the protein data bank^5^. The detection and analysis of proteins and nucleic acids in body fluid (blood, urine, sweat, and saliva), termed “liquid biopsy”, has attracted research in the fields of cancer, cardiovascular diseases, dementia, as well as infectious diseases such as the novel coronavirus disease 2019 (COVID-19)^6–9^. Among the various approaches to chemically quantify proteins^10^, the enzyme immunoassay (EIA) and enzyme-linked immunosorbent assay (ELISA), based on fluorescence or electrochemical signals, are widely used^11,12^. Other analytical methods include mass spectroscopy^13^, electrophoresis^14^, western blotting^15^, and magnetic beads modified with probe molecules^16,17^. In addition, research on nanoscience and nanotechnology have enhanced the detection sensitivity of proteins based on surface plasmon resonance (SPR) in metallic thin films^18^ and localised surface plasmon resonance (LSP) in metallic nanoparticles (NPs)^19^, chemical luminescence^20,21^, and quantum dots^22^. Further, a colorimetric ELISA with the help of the LSP analysis of gold NPs enabled naked-eye detection of the results, greatly enhancing the sensitivity of the method, whereas the detection could take over 6 h due to various assay steps^23^.

Although the copies of nucleic acid molecules (DNA and RNA) can be increased using the polymerase chain reaction (PCR) for the enhancement of assay sensitivity, the number of protein molecules cannot be increased. Therefore, many researchers have relied on fluorescent labelling or other signal enhancements that involve complicated pre-treatment processes. Because the assay time is limited due to these procedures and the low concentration in the observation region, we must develop a strategy beyond conventional approaches. By focusing on guiding samples to the target position, optical tweezers enable us to trap target biomaterials and move them towards the observation region^24^ to detect the intermolecular binding force^25^. Optofluidic approaches have been developed for the detection of small amounts of biological samples with a help of fluidic pressure^26–28^. On the other hand, superradiance from the quantum interaction of LSPs in optically trapped metallic NPs was proposed to enhance optical signals^29^, and the highly sensitive detection of DNA modified on gold NPs was demonstrated with this phenomenon via light-induced hybridisation at the air–liquid interface^30^. The enhanced optical condensation of microscale and nanoscale biomaterials was achieved using photothermal convection with metallic NPs and nanofilms^31–33^. Also, the detection of proteins via shrinkage of photothermally generated bubbles was attempted^34^. Although these reports using photothermal effects provided important aspects for the enhanced detection sensitivity of biomaterials, the major biochemical reactions (e.g. antigen–antibody reaction^35^, biotin–avidin binding^36^) related to proteins are weak against heat; hence, local condensation at the solid–liquid interface, which circumvents this limitation, is strongly desired for their analysis.

**Fig. 1:**
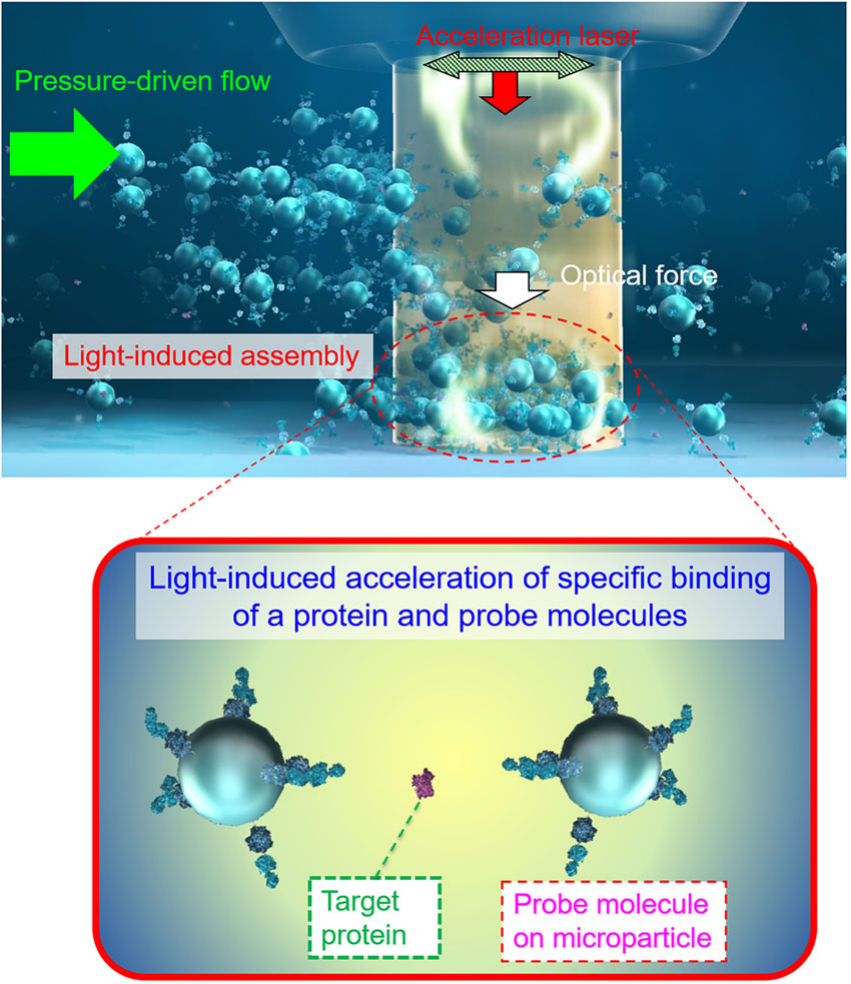
Light-induced acceleration of the interaction among proteins sandwiched between probe-molecule-modified beads. Light-induced acceleration of molecular specific binding mediated by optical force and pressure-driven flow in the confined geometry of the microchannel.

Here, by exploiting the synergetic effect of optical force and fluidic pressure in the microscale narrow space (Fig. 1), we overcome the above-mentioned trilemma (unable to amplify, complex pretreatment, thermal damage), and explore the principle underlying the acceleration of molecular recognition for the rapid high-sensitivity detection of small amounts of specific proteins via achieving a high local concentration, less thermal effect, and less complicated process simultaneously. We attempted to demonstrate the light-induced acceleration of the specific binding of antibody and membrane proteins (CD80^37–39^ or CD9/CD63 fusion protein^40^), which is an example of molecular recognition between proteins, under confinement in a microchannel as narrow as the laser spot in order to greatly increase the collisional probability of target and probe molecules on microparticles.

## Results

### Light-induced acceleration of the specific binding between the antibody and membrane protein

**Fig. 2:**
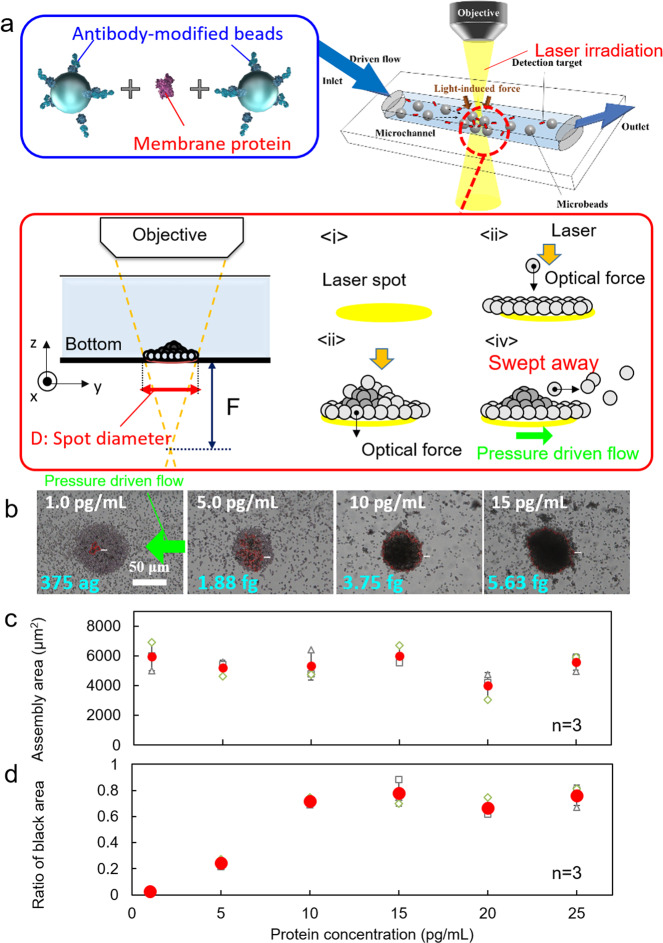
Top-down light-induced acceleration of antigen–antibody reactions with a membrane protein. **a** Schematic diagram of the process of forming a three-dimensional structure by an antigen–antibody reaction with a defocused laser spot diameter. **b** Optical transmission image of the assembled structure of CD80 as the target and probe beads modified with anti-CD80 antibody. The pressure-driven flow is in +*y*-direction, which appears to be inverted in the microscope transmission image. Protein concentration dependence of **c**, total assembly area of the complex of CD80 and probe beads, and **d** ratio of the black area (multi-layered part). In **c** and **d**, error bars indicate standard deviation. Individual data points are shown as small plots.

As the first demonstration of the light-induced acceleration of the specific binding between a protein and host molecule, we targeted membrane protein and pushed antibody-modified microbeads as probe particles from the ceiling of a microchannel to the bottom by the optical force of an irradiated laser to avoid the non-specific adsorption of the particle by automatic washing under pressure-driven flow and gravitational force (Fig. 2a and Supplementary Fig. 1). As the preliminary experiment before the light-induced acceleration of the specific binding between a protein and antibody, we used streptavidin exhibiting the strong binding with biotin (with a dissociation constant *K*_d_ ~ 10^−15^ M) and pushed biotin-modified microbeads as probe particles from below onto the ceiling of a microchannel by the optical force of irradiated laser. The aggregates were obtained even after irradiation with the lower laser power under the help of pressure-driven flow, and the size of the aggregates positively correlated on the concentration of the target protein even in the low concentration region. On the other hand, when laser irradiation was performed below the microchannel in a static fluid, non-specific adsorption between probe particles is prominently observed. There was no correlation between the protein concentration and the size of the obtained aggregates. This may be because there are no transporting effects and no washing effects without pressure-driven flow. These results implied the possibility of highly efficient light-induced acceleration of specific binding of proteins and probe molecules by setting an optimal pressure-driven flow. In this preliminary experiment, a dendritic aggregate was formed to hang from the ceiling owing to the effect of gravity and diffusion-controlled reaction due to the strong binding of streptavidin and biotin. However, such a structure would cause errors in the measurement of the aggregate's cross-sectional area and the aggregates were fragile in the case of antigen-antibody reaction in membrane proteins with low affinity.

Therefore, in Fig. 2, target proteins and antibody-modified microbeads were pressed against the bottom of the channel by laser irradiation from the top after introducing using a syringe pump (laser spot was set to 65 μm below the bottom of the channel), considering the first flat layer of microbeads was used as the binding site for the effective use of gravity. CD80 is a transmembrane protein expressed on antigen-presenting and natural killer cells; it is known to regulate T-cell activation and inactivation^37^. Wild-type CD80 molecules exist in a mixed population of monomeric and dimeric forms, and its affinity with other proteins was reported to show *K*_d_ on the order of 10^−8^–10^−7^ M^38^. The size of the probe particle was selected to be 2 μm in diameter, which exhibits a high scattering efficiency of laser photons at 1064 nm wavelength (Supplementary Fig. 2). Also, we confirmed that the photoemission from a temperature-responsive fluorescent probe is very weak and there is no thermal convection (Supplementary Fig. 3), which indicates small photothermal effect at the wavelength and power of used laser light.

As shown in Fig. 2a, we systematically investigated the aggregated structures when the target protein and probe particles were pressed against the bottom of the microchannel by optical pressure (1–25 pg/mL), where the laser power was set to 530 mW after ×40 objective lens and laser irradiation time was 3 min. Figure 2b shows an optical transmission image of the bottom of the channel captured after the irradiation stopped. The *yz* cross-section of the used microchannel (350 μm × 100 μm), and the flow rate (0.25 μL/min) generated an average flow velocity of 119 μm/s. In Fig. 2b, the area of the first layer (6000 μm^2^) in Fig. 2c (87.4 μm in diameter), is about the same as that of the defocused laser spot diameter (*D* = 68.3 μm) and almost constant regardless of the protein concentration. The circular aggregates (several tens of micrometres in diameter) near the laser-irradiated point in the centre of the snapshot, had distinct black areas. The size of the black area was positively correlated with the target protein’s concentration, and the rate of the black area to the total aggregation area was plotted (Fig. 2d). A clear difference was found between 1–5 and 5–10 pg/mL, where two-tailed *t*-test was used for the evaluation of calibration curve with 0.05 significance level. The black area may be a multilayered structure of antibody-modified beads specifically bound to CD80 by an antigen–antibody reaction accelerated by the optical pressure and pressure-driven flow. As the comparable experiment with ELISA was shown in the previous literature^39^, and the measurement range was 20 pg/mL–20 ng/mL. This means our clarified mechanism can be used for a one- or two-order low concentration range below the limit of detection of ELISA.

Based on these experimental facts, the mechanism underlying the formation of the structure is explained by the process <i>–<iv> in the red box of Fig. 2a. The laser spot is defocused at the bottom of the channel as shown in <i>. When the irradiation starts, the flowing particles are trapped in the laser spot owing to the optical force, as shown in <ii>. Because the probe particles are continuously provided by the pressure-driven flow, the probe particles eventually start to be stacked in multiple layers, as shown in <iii>. When the dispersed antigens (target proteins) cross-link the antibodies modified on the particle surface, they bind to each other via an antigen-antibody reaction. After the irradiation stops, the unbound particles are swept away, and only the specifically bound particles remain as shown in <iv>. These results demonstrate the specific detection of the CD80 protein at a concentration of several pg/mL with two-fold higher sensitivity after only 3 min defocused laser irradiation.

### Efficient light-induced acceleration of protein interactions in the narrow microchannel

**Fig. 3:**
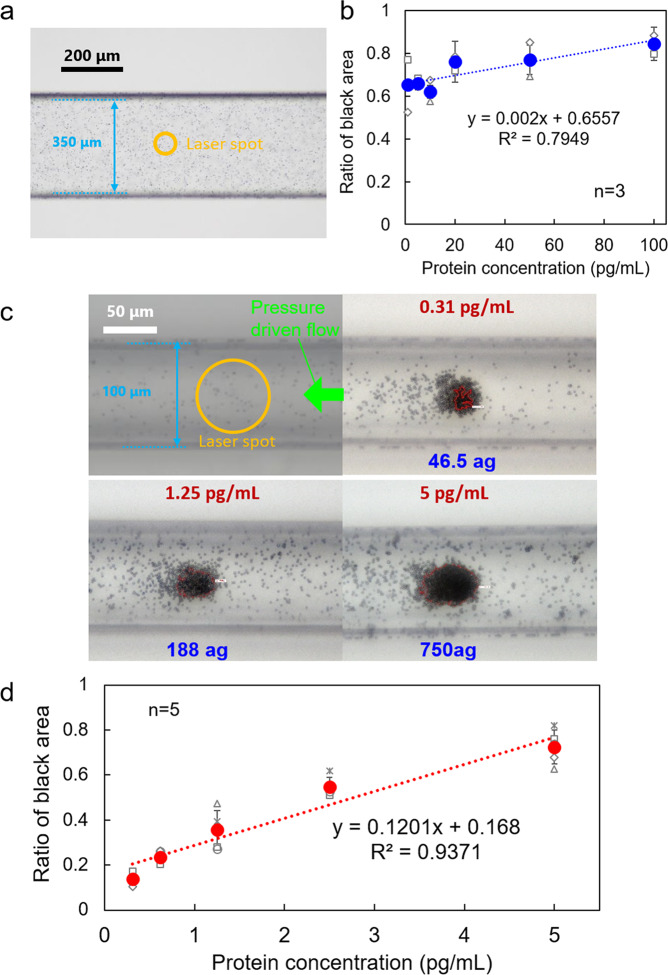
Enhancement of protein-detection efficiency by narrow microchannel comparable to laser spot. **a** Optical transmission image of a wide microchannel (350 μm × 100 μm) and **b** protein concentration dependence of the ratio of the black area representing the assembled structure of the probe beads and CD9/CD63 fusion protein; laser focusing position is adjusted to 65 μm below the bottom of the microchannel and the spot diameter at the bottom is expanded to 68.3 μm (orange circle). **c** Optical transmission image of a narrow microchannel (100 μm × 100 μm) and **d** protein concentration dependence of the ratio of the black area representing the assembled structure of the modified CD9/CD63 fusion protein and probe beads modified with anti-CD63 antibody, where the (laser focusing position and spot diameter correspond to those in **a** and **b**; the size of the orange circle is the same as in **a**). In **b** and **d**, error bars indicate standard deviation. Individual data points are shown as small plots.

In Fig. 3a, as another example of a membrane protein, the CD9/CD63 fusion protein^39^ was introduced into the same-sized microchannel as that used in Fig. 2 (350 μm × 100 μm), together with anti-CD63 antibody-modified microbeads. Also, the laser power was set to 530 mW after ×40 objective lens and laser irradiation time was 3 min. The ratio of the black area gradually increased depending on the protein concentration (Fig. 3b). However, there is a very small difference between 1 and 10 pg/mL with large variability, indicating the low affinity between target protein and antibody. This means that the affinity would be lower than CD80 and further improvement of the detection method was required. Figure 3a shows a snapshot of the bottom of the channel and reveals that a large area around the laser spot (68.3 μm in diameter) is not irradiated in the wide narrow channel (width, 350 μm), and Supplementary Fig. 4 shows the relationship between the laser spot diameter and each channel width. Thus, if we could adjust the laser spot size and channel width to the right, a high-efficiency light-induced assembly could be constructed without wasting the sample.

From these assumptions (Figs. 2b–d and 3a, b), we tried to adjust the relation between laser spot diameter and channel width when the laser beam was focused at 65 μm below the bottom of the channel. When the scale is superimposed on a snapshot of the bottom with a *yz* cross-section of 100 μm × 100 μm, it covers most of the channel width with a small gap at the edge. Under these conditions, the laser power was set to 265 mW after ×40 objective lens, and the laser was irradiated for 3 min by setting the focal point at 65 μm below the bottom of the channel along the *z*-axis. The optical transmission images at respective concentrations (Fig. 3c) were captured from the bottom of the channel after stopping the laser irradiation, and the size of the assembled structure was analysed using the same method as that in Fig. 2d. For each snapshot, the ratio of the black region to the area of the total region was calculated (Fig. 3d). The *yz* cross-section of the microchannel used here is 100 μm × 100 μm, and the volume flow rate of the syringe pump is 0.1 μL/min (average flow velocity of 167 μm/s). The linearity of the graph (*R*^2^) exceeded 0.93, and a clear calibration curve was drawn. The slope was positive and showed a significant difference in all intervals (two-tailed *t*-test was used for the evaluation of the calibration curve with a 0.05 significance level.). On the other hand, when we introduced anti-CD63-antibody-modified beads and CD80, we confirmed that the ratio of the black area did not correlate with the concentration (Supplementary Fig. 5). These results indicate that CD9/CD63 fusion protein was specifically detected by the light-induced acceleration of antigen-antibody reaction in microflow in Fig. 3d since there is no correlation with the concentration of CD80 as the mismatched target. There is a possibility that the black area formed independently of CD80 by non-specific binding with the other components in the solution.

In particular, we revealed the conditions required to improve the detection limit (detection range: 0.31–5.0 pg/mL) for CD9/CD63 fusion proteins and the detection time within a few minutes, which is one order of magnitude lower than that obtained via the conventional ELISA method (detection range: 3.1–200 pg/mL) as shown in Supplementary Figs. 6 and  7. Considering that the ELISA takes ~6 h for a total detection time, we successfully achieved the ~100-fold faster detection of very small amounts of proteins by light-induced acceleration. From the lowest detection limit, we can estimate that attogram-level detection (46.5 ag) is possible corresponding to the zmol-level sensitivity, where 2556 proteins pass through the channel in a 300-nL sample after 3 min of laser irradiation (a molecular weight of CD9/CD63 fusion protein: 21.9 kDa; volume flow rate: 0.1 μL/min). The average total area of the assembled particles at the bottom is 1420 μm^2^ corresponding to 452 particles for Fig. 3c, with 62 particles forming a multilayered black region (enclosed within the red curve) via light-induced antigen–antibody reactions. If 2556 proteins would be sandwiched between 62 pairs of probe particles, ~41 proteins would be trapped between each pair of probe particles in the black region, implying the detection of sub-zmol proteins with a simple optical manner. On the other hand, although isolated CD9/CD63 fusion proteins could be detected by ELISA with a pair of anti-CD63 antibody and anti-CD9 antibody (Supplementary Fig. 6), they could not be detected by ELISA with a pair of anti-CD63 antibodies (Supplementary Fig. 7). From these experimental facts, we consider that CD9/CD63 fusion proteins would form an aggregate with a specific binding site on the outside and they would be sandwiched between the anti-CD63 antibody-modified beads under laser irradiation as shown in Supplementary Fig. 8.

### Theoretical calculation of microflow-type light-induced acceleration

**Fig. 4:**
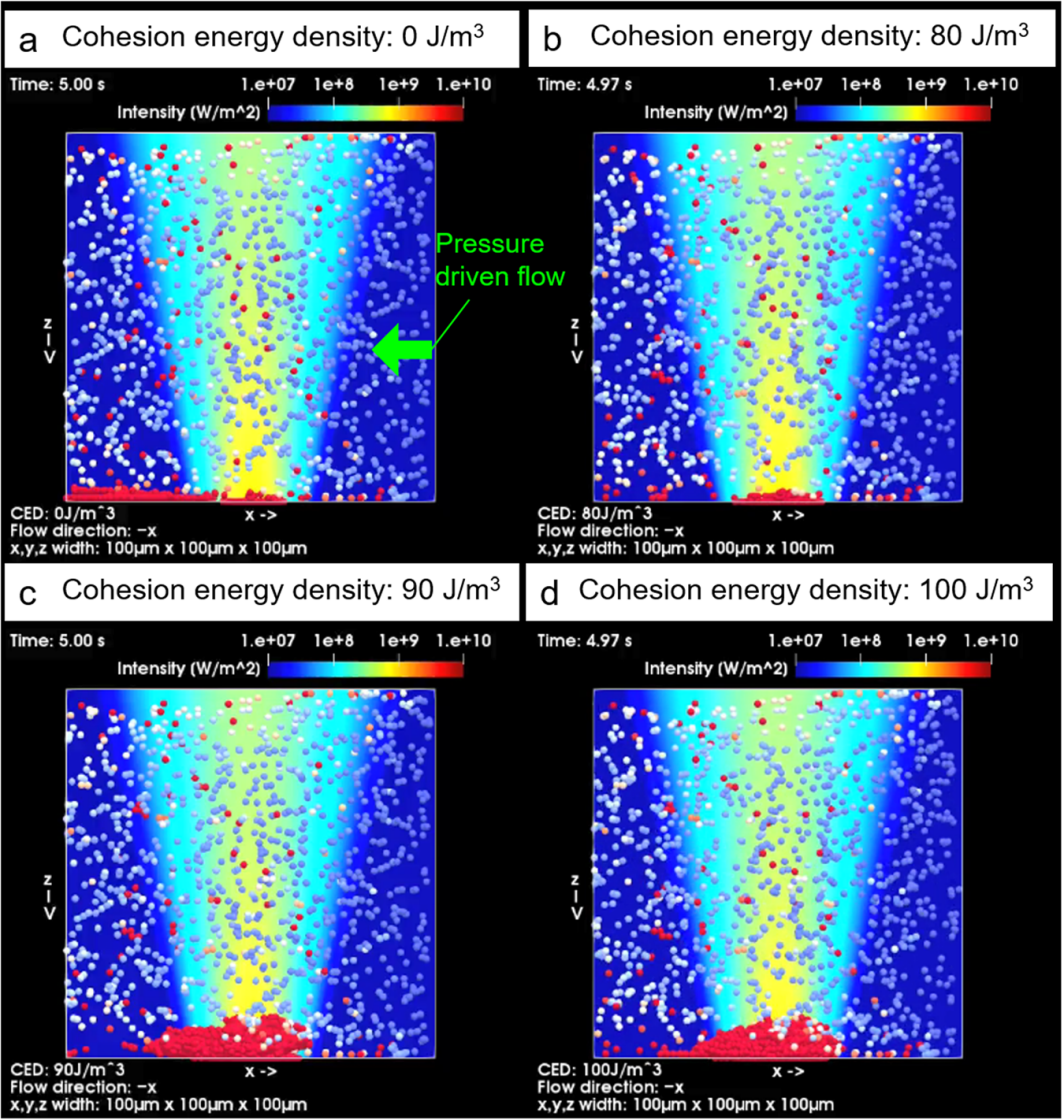
Theoretical simulations of light-induced acceleration of antigen–antibody reaction in a microflow. Calculated results of light-induced assembly of microparticles by optical pressure in microflow channel for different cohesion energy densities between antigen and antibody on each bead assuming the different antigen concentrations (volume flow rate: 0.1 μL/min, Laser power: 530 mW, laser spot was set to 65 μm below the bottom of the channel). In each panel, cohesion energy was assumed to be **a**: 0 J/m^3^, **b**: 80 J/m^3^, **c**: 90 J/m^3^, **d**: 100 J/m^3^. The colour of the particle indicates the time elapsed since the particle appeared from the inlet (blue: 0 s, white: 1 s, red: 2 s). See also Supplementary Movies 1 and 2.

Figure 4 shows a simulation where the channel width was narrow (100 μm × 100 μm) to discuss the essence of the phenomena. By varying the binding force between the beads (changing cohesion energy density *E*_c_) to investigate the effect of antigen–antibody reaction, it was qualitatively clarified that the stronger binding force can generate a multilayered structure easier (see also Supplementary Movies 1 and 2).

Under the paraxial approximation, the acceleration laser was assumed to be Gaussian beam with wavelength *λ* = 1064 nm with power *P* = 530 mW, and the radius of beam waist is set to *w*_0_ = 1.3 μm with ×40 objective lens (NA = 0.6) similarly to the experiment. The *z*-position of the focal point from the bottom of the microchannel is assumed to be F as shown in the red box of Fig. 2a (65 μm below the bottom). The Navier–Stokes equation can be transformed into the Poisson equation by considering the steady state of an incompressible Newtonian fluid in a rectangular region such as a microchannel cross-section moving in the depth direction. The analytical solution of the flow velocity is obtained by solving this equation under the no-slip boundary condition at the wall surface and the introduced mean flow velocity as a volumetric flow rate 0.1 μL/min of water into a region with a cross-sectional area of 100 μm × 100 μm as in the experiment shown in Fig. 3c, d. Furthermore, to calculate the dynamics of the probe particles under light-induced force and pressure-driven flow, the discrete element model of OpenFOAM v7^41^ was used with a modification of adding the forces. The direction of a pressure-driven flow corresponds to the experimental microscope transmission image. The following equation of motion was evaluated using the Euler method:

1 $${m}_{{{{{{\rm{p}}}}}}}\frac{{{{{{{\rm{d}}}}}}}{{{{{{\bf{u}}}}}}}_{{{{{{\rm{p}}}}}}}}{{{{{{{\rm{d}}}}}}}t}={{{{{{\bf{F}}}}}}}_{{{{{{\rm{C}}}}}}}+{{{{{{\bf{F}}}}}}}_{{{{{{\rm{D}}}}}}}+{{{{{{\bf{F}}}}}}}_{{{{{{\rm{G}}}}}}}+{{{{{{\bf{F}}}}}}}_{{{{{{\rm{R}}}}}}}+{{{{{{\bf{F}}}}}}}_{{{{{{\rm{opt}}}}}}},$$

where *m*_p_ is the mass and **u**_p_ is the velocity vector of the probe particle. **F**_C_ is the contact force as the function of cohesion energy density *E*_C_ [J/m^3^], which changes the binding behaviour depending on the amount of target protein and affinity with probe molecule. (The higher the *E*_C_, the stronger the binding force between particles is assumed.) **F**_G_ is gravity, **F**_D_ is the drag force, **F**_R_ is the random force associated with Brownian motion, and **F**_opt_ is the light-induced force^29,42,43^. The flow in the channel is also numerically solved and drives the particles via **F**_D_, where the boundary condition of the pressure difference 47.424 mPa between inlet and outlet with 100 μm length was determined from the analytical solution. As for the contact between the probe particles and the channel wall, we assumed a situation where the particles are fixed when they are strongly pressed against the wall. In order to discuss the essence in a simple situation, **F**_opt_ was assumed to be represented by an approximated form^43^ as the sum of the gradient force proportional to∇|**E**_inc_|^2^ and dissipative force proportional to Poynting vector of the irradiated Gaussian beam **S** as follows:

2 $${{{{{{\bf{F}}}}}}}_{{{{{{\rm{opt}}}}}}}=\frac{1}{4}{\alpha }_{{{{{{\rm{opt}}}}}}}\nabla {|{{{{{{\bf{E}}}}}}}_{{{{{{\rm{inc}}}}}}}|}^{2}+\frac{{n}_{{{{{{\rm{f}}}}}}}{\sigma }_{{{{{{\rm{ext}}}}}}}}{c}{{{{{\bf{S}}}}}},\,{\alpha }_{{{{{{{\rm{opt}}}}}}}}=3{V}_{{{{{{\rm{p}}}}}}}{\varepsilon }_{{{{{{\rm{f}}}}}}}{\varepsilon }_{0}\frac{{\varepsilon }_{{{{{{\rm{p}}}}}}}-{\varepsilon }_{{{{{{{\rm{f}}}}}}}}}{{\varepsilon }_{{{{{{\rm{p}}}}}}}+2{\varepsilon }_{{{{{{{\rm{f}}}}}}}}},$$

where *α*_opt_ is the polarizability of probe particle, **E**_inc_ is the incident light electric field, *n*_f_ is the refractive index of surrounding medium, and σ_ext_ is the extinction cross-section of the probe particle with anisotropic factor obtained by Mie scattering theory assuming the plane wave irradiation. *α*_opt_ is given by the volume *V*_p_ of the probe particle (2 μm in diameter), the refractive index of probe particle is *n*_p_ = *ε*_p_^1/2^ = 1.57, and the refractive index of the medium water is *n*_f_ = *ε*_f_^1/2^ = 1.33. *σ*_ext_ was numerically evaluated as 9.07 μm^2^, and the light intensity is given by *I* = *P*/*A* = *c*|**S**|=*ε*_f_|**E**_inc_|^2^/2 with the laser spot area *A* as *I* = 41.7 kW/cm^2^ at the ceiling and *I* = 150 kW/cm^2^ at the bottom of the microchannel, respectively (*ε*_0_ is the dielectric constant of the vacuum, *c* is the speed of light in vacuum). To take into account the effect of attenuation in the medium phenomenologically, the factor based on Lambert–Beer was multiplied to **S** and ∇|**E**_inc_ | ^2^, while the *z*-derivative of this factor was not considered. In this case, the dissipative force was evaluated as 16.8 pN at the ceiling and 60.4 pN at the bottom, respectively. The concentration of probe particles was initially 1.148 × 10^9^ particles/mL, and the particles were randomly placed at an initial condition. The simulation was run for up to 5 s, and it was assumed that during this time, particles flowed in from the inlet with the product of the particle concentration and the volume flow rate, where the particles reaching the outlet disappeared. The colour of the particle indicates the time elapsed since the particle appeared from inlet (blue: 0 s, white: 1 s, red: 2 s). In other words, the redder the particle in the laser spot, the longer it has been strongly bound (Fig. 4a–d, Supplementary Movies 1 and 2). As the higher cohesion energy, the thicker optically assembled multilayered structures in red colour were produced by laser irradiation and maintained for a long time. Although the antigen–antibody-binding strength is uniquely determined by the combination of a pair of antigen and antibody, the higher cohesion energy in these simulations indicates that the greater amounts of antigens were sandwiched between antibody-modified microparticles under light-induced acceleration. The multilayered structure formation would be mediated by such a process. If the laser spot size becomes smaller as shown in Supplementary Fig. 9, the optical force gets stronger but the microparticles were assembled in the narrow region. The assembled structure gets larger by optical force, but the aggregate was swept away by the pressure-driven flow in the case of a lower cohesion energy density 80 J/m^3^. Moreover, as the laser power becomes lower in Supplementary Fig. 10, the light-induced assembled structure gets smaller and more stable for the pressure-driven flow even in the case of lower cohesion energy 80 J/m^3^. On the other hand, the flow rate gets lower, and the height of assembled structure gets larger but the most part was swept away in the case of 80 J/m^3^ (Supplementary Fig. 11). These simulation results qualitatively explain that there is an optimum condition of laser and fluid flow for the highly efficient light-induced acceleration of antigen–antibody reactions. While these calculation results are based on the simple model, they can well explain the experimental results on the assembly phenomena of microparticles by the light-induced antigen-antibody reactions under the confined geometry of microflow in Figs. 2 and 3.

## Discussion

We clarified the guiding principle of the light-induced acceleration of the specific binding between the target protein and probe molecules modified on beads under optical force and fluidic pressure by tuning the laser irradiation area comparable to that of the microscale space. Remarkably, 100-fold higher sensitivity and 100-fold faster specific detection of proteins in 1/100 sample liquid was achieved under the optically accelerated antigen–antibody reaction using 3 min laser irradiation. In addition, we can omit any pre-treatment processes, incubation and washing at the light-induced detection of antigen if the antibody-modified probe microparticles are prepared in advance and stored in a refrigerator. This means the dramatic improvement of detection limit and quantitative measurement is achievable even in a small number of biological samples with a simple process. In particular, our finding on the detection of low-abundance membrane proteins presented here could pave the way for the innovative high-throughput analysis of nanoscale biomaterials such as extracellular vesicles with membrane proteins on their surface (exosomes, microvesicles, etc.) as a promising biomarker of cancer, viruses with unique surface proteins (SARS-CoV-2, influenza, etc.), various kinds of biomolecules related to other diseases, and allergy-related substances.

## Methods

### Microflow-type light-induced acceleration system

The optical system used for the experiment in the light-induced acceleration of membrane protein and antibody-modified probe microparticle is shown in Supplementary Fig. S1. An upright optical microscope (ECLIPSE Ni-E, Nikon, Japan) was used. 60 µL of sample liquid (mixture of the dispersion liquid of membrane protein: 30 μL and dispersion liquid of antibody-modified microparticles: 30 μL) was introduced into a glass syringe (Micro Syringe 1725TLL Lure Lock 21120, 250 µL, Hamilton, USA), and the volume flow rate was controlled by a syringe pump (FUSION TOUCH 200, Isis, Japan). The direction of pressure-driven flow (+*y*-direction) appears to be inverted in the transmission image due to the optical system of our microscope (both the *x*- and *y*-directions are inverted). Thereafter, the near-infra-red continuous wave laser of 1064 nm wavelength (FLS-1064-2000F, Sigma Koki, Japan) was introduced from a backport adapter (LMS-M1064-20001S/LN; Sigma Koki, Japan), and focused using an objective (CFI S Plan Fluor ELWD 40XC, 0.6 NA) on the bottom of the microchannel. A hydrophilic wide microchannel (width 350 µm × height 100 µm BS-21102, Sumitomo Bakelite, Japan) was used for the experiment of CD80, and a hydrophilic narrow microchannel (width 100 µm × height 100 µm MiniLuer 0144 COC, ASICON, Japan).

The laser spot diameter was 2.6 µm at the focal point and the laser power was determined with a laser power meter (UP17O-H5 and TUNER; Gentec Electro-Optics, Canada), and the spot position was controlled for the defocus condition to generate optical pressure over the wide region. The optical transmission image of the assembled structure was recorded by a CCD camera (DS-Filc-L3, Nikon, Japan) under the irradiation of white light from a halogen lamp after 180 s of laser irradiation. The cross-sectional areas of the assembled structure and multi-layered structure (black region) were analysed with commercial software (NIS Elements, Nikon, Japan). *t*-test was used for the evaluation of significant differences in calibration curves.

### Sample preparation for membrane protein (CD80) and antibody-modified probe particle

Membrane protein CD80 was used as the target, and biotin-labelled anti-CD80 antibody was used as the probe molecule (both were provided by Sysmex Corporation)^39^. The stock solution of CD80 dispersion was 250 μg/mL and diluted to the target concentration for the experiment using phosphate buffer as the solvent. The used phosphate buffer was self-made by mixing disodium hydrogen phosphate (197-02865, Wako Pure Chemical Industries) and sodium dihydrogen phosphate (197-09705, Wako Pure Chemical Industries) at 10 mM, pH = 7.0.

Then, the anti-CD80 antibody-modified streptavidin-labelled polystyrene latex beads (2 μm in diameter, 24160, Polysciences) were used as probe microparticles. The used phosphate buffer was the same as that used for the CD80 dilution. First, streptavidin-labelled polystyrene particles were diluted to 1.148 × 10^9^ particles/mL with 10 mM phosphate buffer. Biotin-labelled anti-CD80 antibody (undiluted concentration 1 mg/mL) was diluted to 20 μg/mL in 10 mM phosphate buffer. Equal amounts of the prepared Latex bead solution and anti-CD80 solution were mixed, and the mixture was placed in an incubator at 41.7 °C for 1 h. The beads were removed from the incubator and washed five times by centrifugation (10,000×*g*, 5 min) with 10 mM phosphate buffer. The prepared anti-CD80 antibody-modified beads were stored refrigerated at 4 °C.

### Sample preparation for membrane protein (CD9/CD63 fusion protein) and antibody-modified probe particle

CD9/CD63 fusion protein was used as the target and a biotinylated anti-CD63 antibody was used for the modification of polystyrene latex microparticle (2 μm in diameter, 24160, Polysciences) after the confirmation of the calibration curve by changing the amounts of target proteins. Our used CD9/CD63 fusion protein and biotin-labelled anti-CD63 antibody were included in the CD9/CD63 ELISA kit (EXH0102EL, Cosmo Bio Co., Ltd)^40^. The stock concentration of CD9/CD63 fusion protein is 2 μg/mL, and a small amount of the volume of 200 μL was diluted to the target concentration used in the experiment using phosphate buffer 10 mM as a solvent (as in the case of CD80). Streptavidin-labelled polystyrene latex particles were diluted to 1.148 × 10^9^ particles/mL in 10 mM phosphate buffer (same as that prepared for CD80).

Biotin-labelled anti-CD63 antibody (Cosmo Bio Inc., SHI-EXO-M02-B) was undiluted at a concentration of 1 mg/mL and diluted to 20 μg/mL with 10 mM phosphate buffer. The diluted biotin-labelled anti-CD63 was washed five times by centrifugation (10,000 × *g*, 5 min) with 10 mM phosphate buffer. Equal amounts of the prepared Latex bead solution and anti-CD63 solution were mixed and placed in an incubator at 41.7 °C for 1 h. The beads were removed from the incubator and washed five times by centrifugation (10,000 × *g*, 5 min) with 10 mM phosphate buffer. After preparation, the mixture was stored refrigerated at 4 °C.

### Statistics and reproducibility

*A* two-tailed *t*-test was used for the evaluation of the calibration curve with a 0.05 significance level. All the experiments were successfully replicated, and the number of replicates is specified for each experiment.

### Reporting summary

Further information on research design is available in the Nature Research Reporting Summary linked to this article.

## Supplementary information

Supplementary Information

Description of Additional Supplementary Files

Supplementary Movie 1

Supplementary Movie 2

Supplementary Data 1

Reporting Summary

## Data Availability

All data needed to evaluate the conclusions in the paper are presented in the paper and/or the Supplementary Materials. Additional data related to this paper may be available from the corresponding authors upon reasonable request. Source Data are available in Supplementary Data 1.
