# Supplementary material for: Attogram-level light-induced antigen-antibody binding confined in microflow
Source: Commun Biol. 2022 Oct 6;5:1053. doi: 10.1038/s42003-022-03946-0 (PMC9537419; doi:10.1038/s42003-022-03946-0)
Supplement: Supplementary file 1 — Supplementary Information [file 42003_2022_3946_MOESM1_ESM.pdf]

SUPPLEMENTARY INFORMATION

Attogram-level Light-induced Antigen-antibody Binding Confined in Microflow

Takuya Iida[1,2,5,6,8*], Shota Hamatani[1,2,3,5,6,7], Yumiko Takagi[1,2,5,6], Kana Fujiwara[1,2,3,5,6,7], Mamoru Tamura[2,4,6], and Shiho Tokonami[2,3,6,7,8*]

[1] Department of Physical Science, Graduate School of Science, Osaka Prefecture University, 1-2 Gakuencho, Nakaku, Sakai, Osaka 599-8570, Japan

[2] Research Institute for Light-induced Acceleration System (RILACS), Osaka Prefecture University, 1-2 Gakuencho, Nakaku, Sakai, Osaka 599-8570, Japan

[3] Department of Applied Chemistry, Graduate School of Engineering, Osaka Prefecture University, 1-2 Gakuencho, Nakaku, Sakai, Osaka 599-8570, Japan

[4] Graduate School of Engineering Science, Osaka University, 1-3 Machikaneyama-cho, Toyonaka, Osaka 560-8531, Japan.

[5] Present address: Department of Physics, Graduate School of Science, Osaka Metropolitan University, 1-2 Gakuencho, Nakaku, Sakai, Osaka 599-8570, Japan

[6] Present address: Research Institute for Light-induced Acceleration System (RILACS), Osaka Metropolitan University, 1-2 Gakuencho, Nakaku, Sakai, Osaka 599-8570, Japan

[7] Present address: Department of Applied Chemistry, Graduate School of Engineering, Osaka Metropolitan University, 1-2 Gakuencho, Nakaku, Sakai, Osaka 599-8570, Japan

[8] These authors contributed equally to this work.

*Corresponding authors: t-iida@omu.ac.jp (T.I.), tokonami@omu.ac.jp (S.T.)

Movie Legends:

Please see also the detail in the main text.

Supplementary Movie 1. Video of theoretical result of light-induced assembly of microparticles by optical pressure in microflow channel, where the interparticle biding force is neglected (Cohesion energy density: 0 J/m^3, Volume flow rate: 0.1μL/min, Laser power: 530 mW, Focal point from the bottom of microchannel (F): 65 μm). This video corresponds to Fig. 4a.

Supplementary Movie 2. Video of theoretical result of light-induced assembly of microparticles by optical pressure in microflow channel, where the interparticle biding force is strong (Cohesion energy density: 100 J/m^3, Volume flow rate: 0.1μL/min, Laser power: 530 mW, Focal point from the bottom of microchannel (F): 65 μm). This video corresponds to Fig. 4d.

Supplementary Figures:

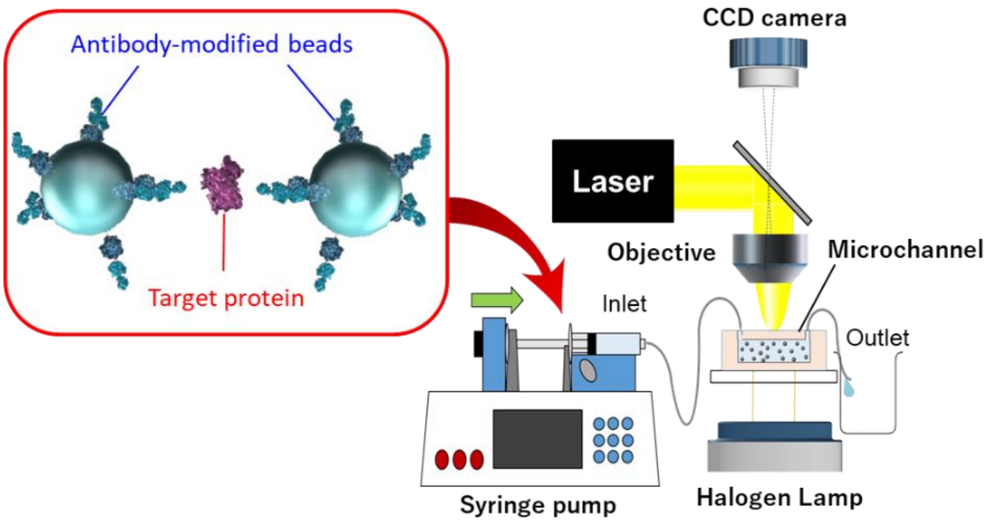

Supplementary Figure 1 | Schematic diagram of the optical systems for microflow-type light-induced acceleration. The optical system for light-induced acceleration of antigen-antibody reactions with upright optical microscope.

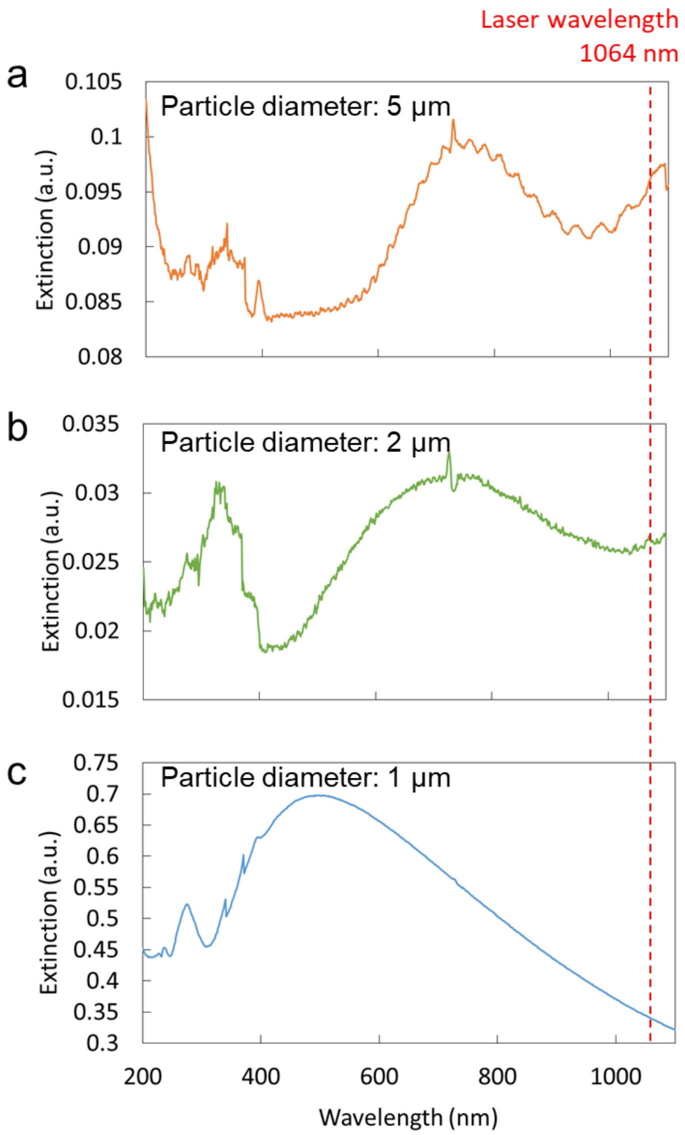

Supplementary Figure 2 | Extinction spectra of polystyrene microparticles with different sizes. a, particle diameter: 5 μm (undiluted concentration: 7.40×10^8 particles/mL), **b,** particle diameter: 2 μm (undiluted concentration: 2.30×10^9 particles/mL), and **c,** particle diameter: 1 μm (undiluted concentration: 9.30×10^{10} particles/mL). The amount of each dispersion liquid was 5 μL for each sized microparticle. These spectra were measured by UV-vis spectrometer (V-630BIO, JASCO Corporation).

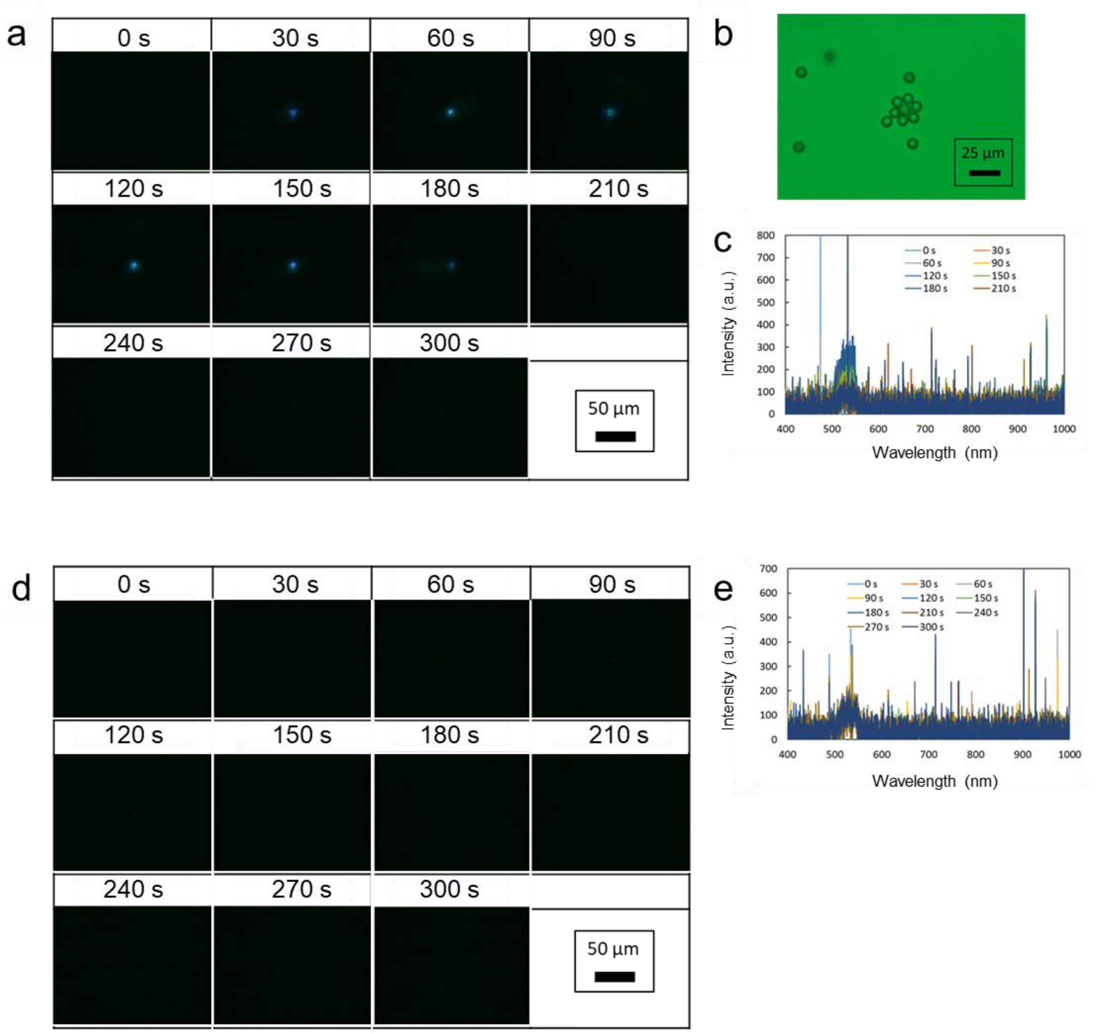

Supplementary Figure 3 | Evaluation of thermal effects of laser irradiation on probe particles at solid-liquid interface in microchannel. a, Temporal change of fluorescence image of a mixture of 5um diameter beads and temperature-responsive fluorescent probe (Particulate Thermoprobe, FDV-0003, funakoshi.inc, excitation wavelength 460 nm, fluorescence wavelength 560 nm). This fluorescent probe strongly emits 560 nm green fluorescence beyond 32 ˚C. **b,** Transmission image of a polystyrene particle with a diameter of 5 μm observed in bright field. **c,** Temporal change of local fluorescence spectra corresponding to **a**. **d,** Temporal changes of fluorescence image of a mixture of beads with a diameter of 2 μm and a temperature-responsive fluorescent probe irradiated by laser, and of **e,** local fluorescence spectra. In both cases, experimental conditions are similar to Fig. 2 and Fig. 3 in the main text, where the measurements were performed using a 40x objective lens with a defocused 0.5 W laser of 1064 nm wavelength.

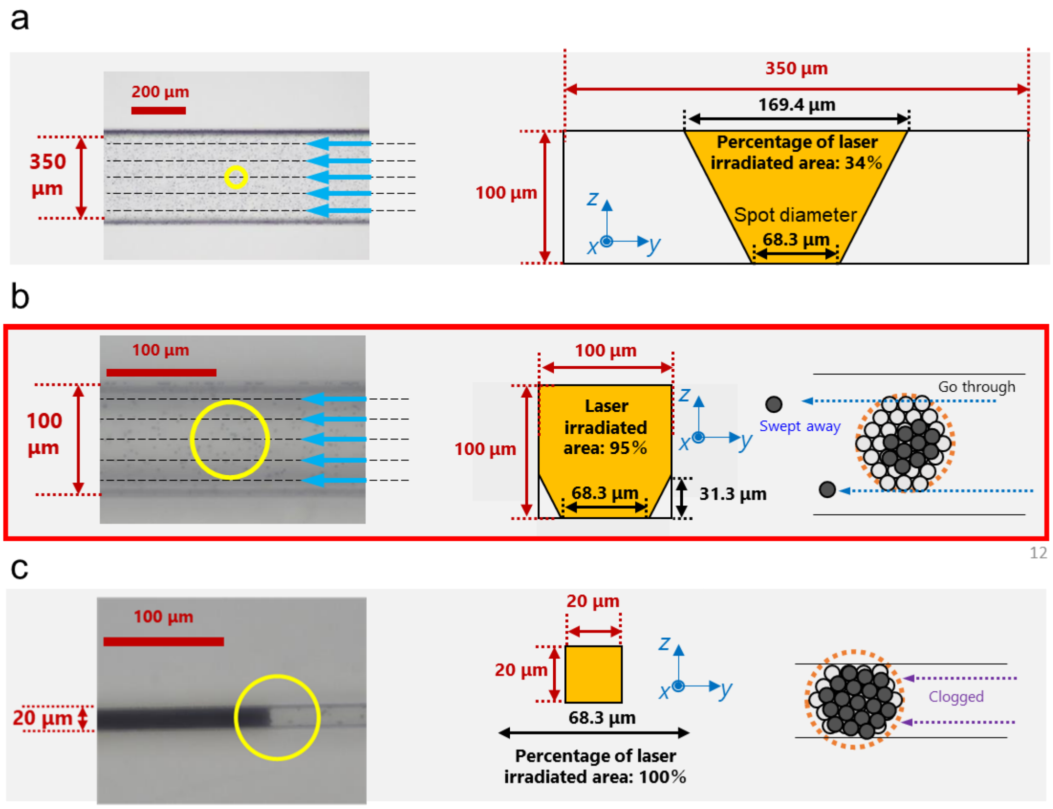

Supplementary Figure 4 | Optimization of the relationship between channel width and spot size for light-induced acceleration. a, When the channel width is much wider than the laser spot diameter, the major part of probe particles was flowed away and the efficiency of light-induced acceleration is low. **b,** When the channel width is comparable to the laser spot diameter, the major part of probe particles passed through the laser spot and highly efficient light-induced acceleration of specific binding can be expected avoiding non-specific binding with sweeping effect at near the channel walls. **c,** When the channel width is much narrower than the laser spot diameter, probe particles were clogged with probe particles by the optical pressure at high probability.

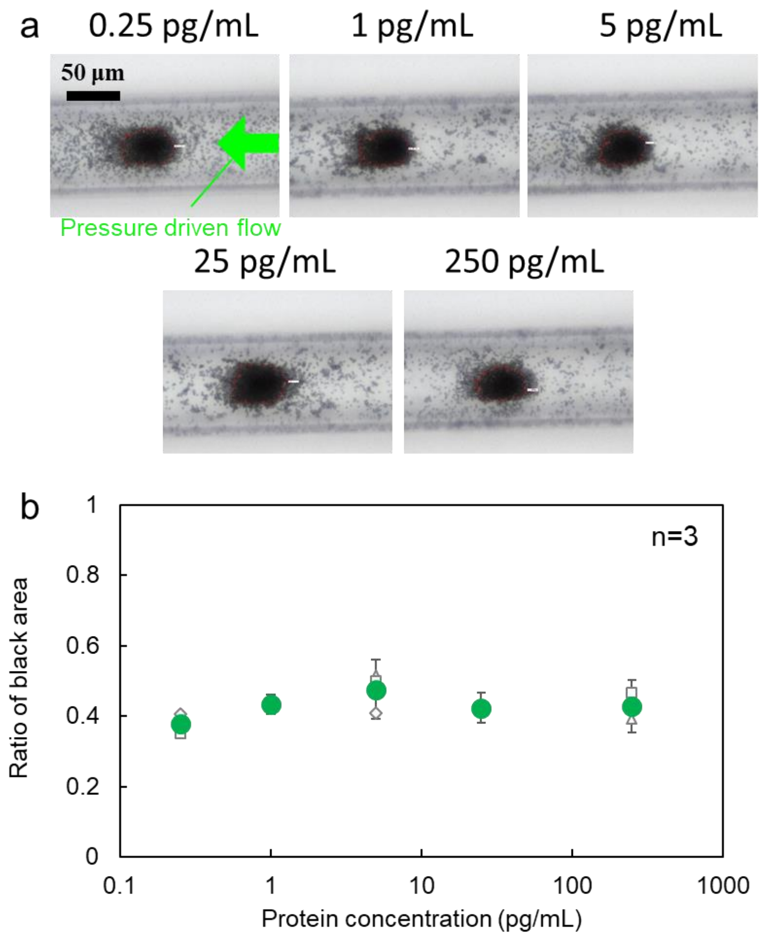

Supplementary Figure 5 | Optical condensation for mismatched condition (CD80 and probe particles modified with Anti-CD63 antibody). a, Optical transmission images of assembled structure of probe particles and CD80 as mismatched target under the same experimental conditions as those of Fig.3c-d. **b,** Protein concentration dependence of ratio of the black area assembled structure of probe beads and CD80. In **b**, error bars indicate standard deviation. Individual data points are shown as small plots.

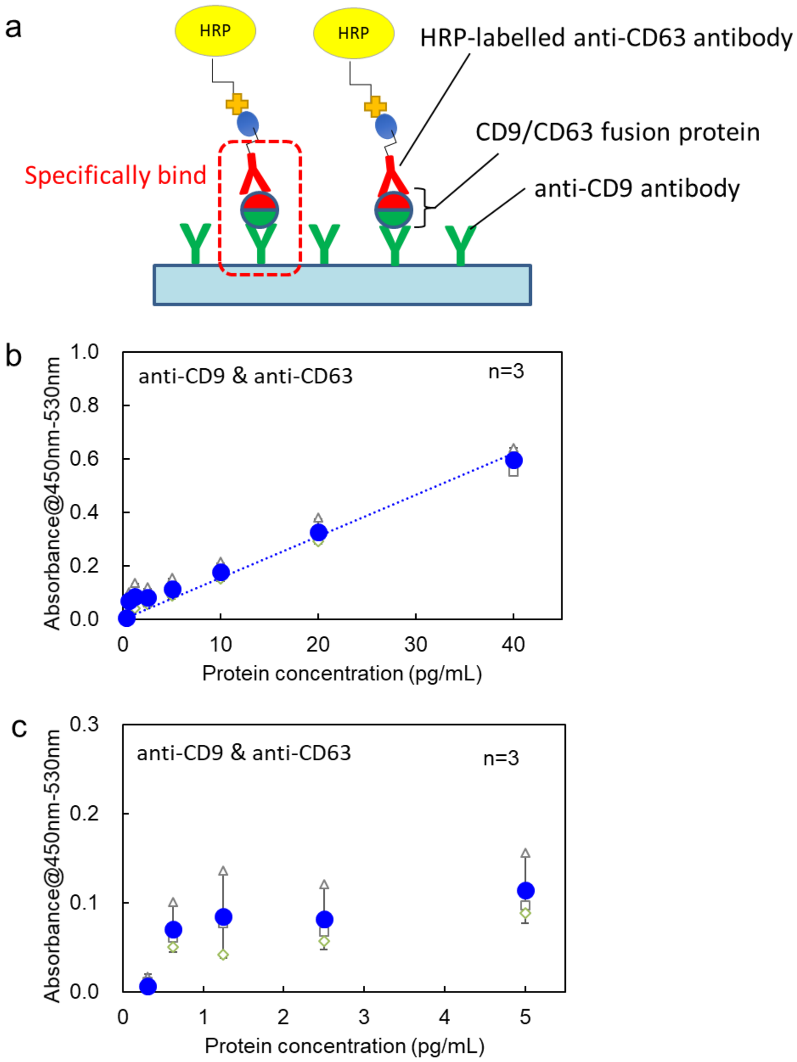

Supplementary Figure 6 | ELISA measurement of CD9/CD63 fusion protein sandwiched by anti-CD9 antibody and anti-CD63 antibody. a, The schematic image indicating that the concentrations in dilution series of CD9/C63 fusion protein were measured according to the protocol of CD9/CD63 ELISA kit (EXH0102EL, Cosmo Bio Co., Ltd), where anti-CD9 antibodies were modified on the bottom of each well as the capture antibody. 100 µL of dilution liquid of CD9/C63 fusion protein with each concentration was added, stirred and incubated for 2 hours. After washing three times with washing buffer, the biotinylated-anti-CD63 antibody (SHI-EXO-M02-B, Cosmo Bio Co., Ltd) was reacted with streptavidin-labelled HRP and added to be incubated for 2 hours. After washing three times, chromogenic substrate was added and incubated for 20 minutes. Finally, stop solution was added, and the absorbance of each microwell was measured at the wavelength of 450nm in each microwell by a plate reader (ARVO X3, PerkinElmer, USA). **b,** the result of ELISA in the high concentration range and **c**, enlarged data in the low concentration range. Error bars indicate standard deviation. Individual data points are shown as small plots.

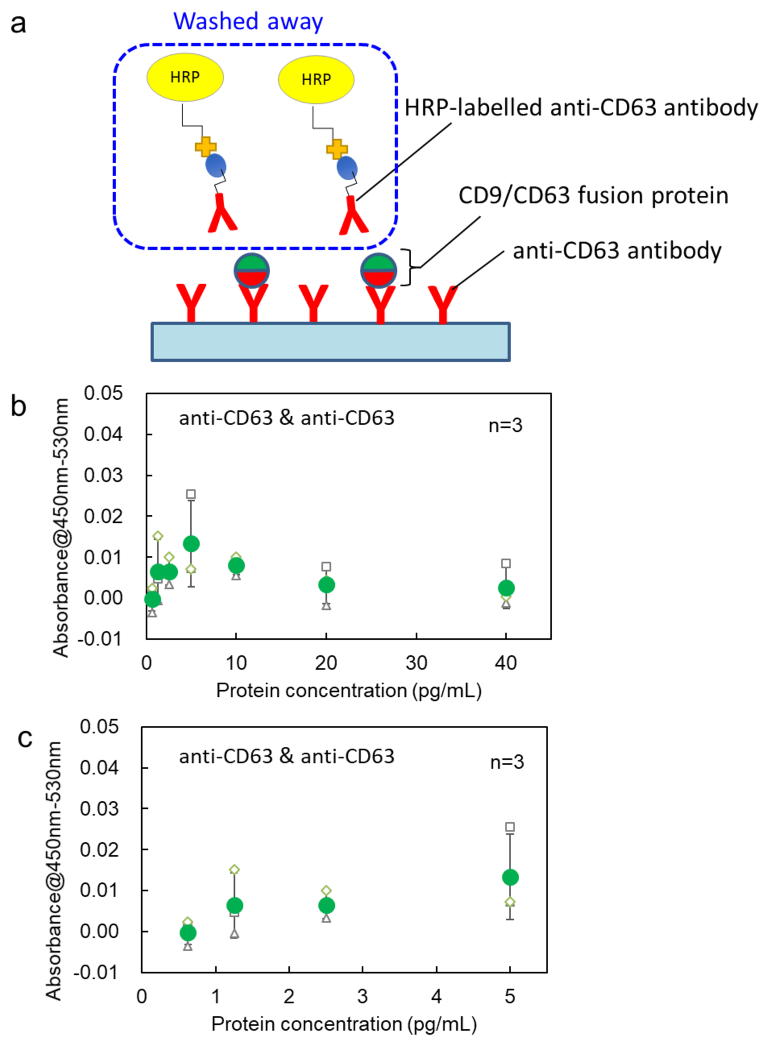

Supplementary Figure 7 | ELISA measurement of CD9/CD63 fusion protein sandwiched by anti-CD63 antibody on the bottom of the well and anti-CD63 antibody. a, The schematic image indicating that the concentrations in dilution series of CD63/C9 fusion protein were measured by the well plate modified with anti-CD63 antibody in the ELISA Kit (FUJIFILM, 290-83601). Assay buffer for dilution was PBS (pH7.2-7.4) with 1% BSA, and washing buffer was PBS (pH7.2) with 0.05% polyoxyethylene(20) sorbitan monolaurate. 100 µL of dilution liquid of CD9/C63 fusion protein with each concentration was added, stirred and incubated for 2 hours. After washing three times with buffer solution, the same biotinylated-anti-CD63 antibody as that in Supplementary Figure 6 (SHI-EXO-M02-B, Cosmo Bio Co., Ltd) was added to be incubated for 2 hours. After washing three times, streptavidin-labelled HRP was added there and incubated for 30 minutes. After washing three times, chromogenic substrate was added and incubated for 30 minutes. Finally, stop solution was added and the difference of absorbance was measured at the wavelengths of 450nm and 530 nm in each microwell by a plate reader (ARVO X3, PerkinElmer, USA). **b,** the result of ELISA in the high concentration range and **c,** enlarged data in the low concentration range. Error bars indicate standard deviation. Individual data points are shown as small plots.

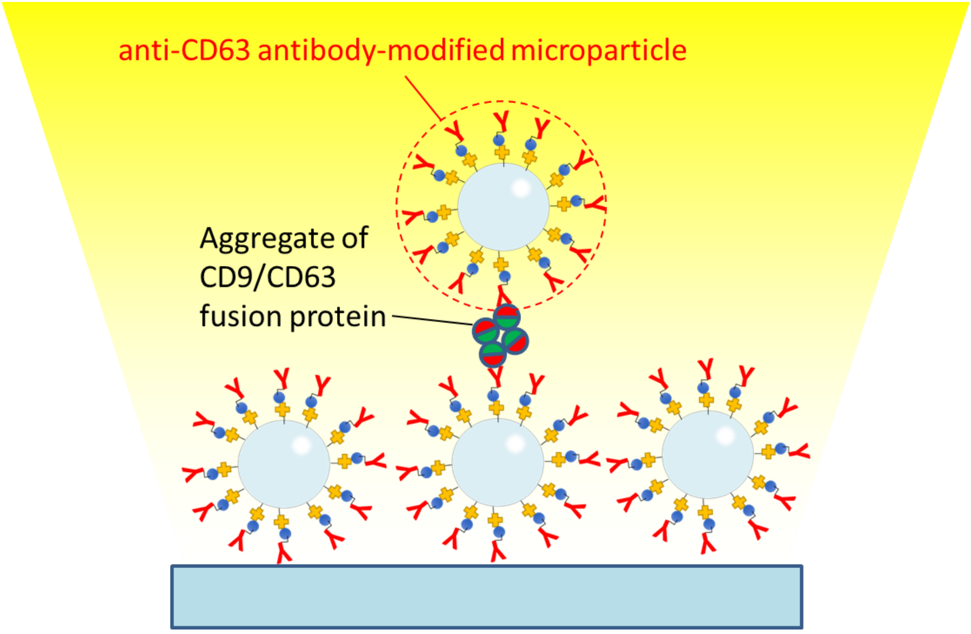

Supplementary Figure 8 | Light-induced acceleration of CD9/CD63 fusion protein and anti-CD63 antibody-modified microparticles. Conceptual image of the assumed model in light-induced detection of CD9/CD63 fusion protein expected from experimental results in Fig. 3, Supplementary Figure 6 and Supplementary Figure 7.

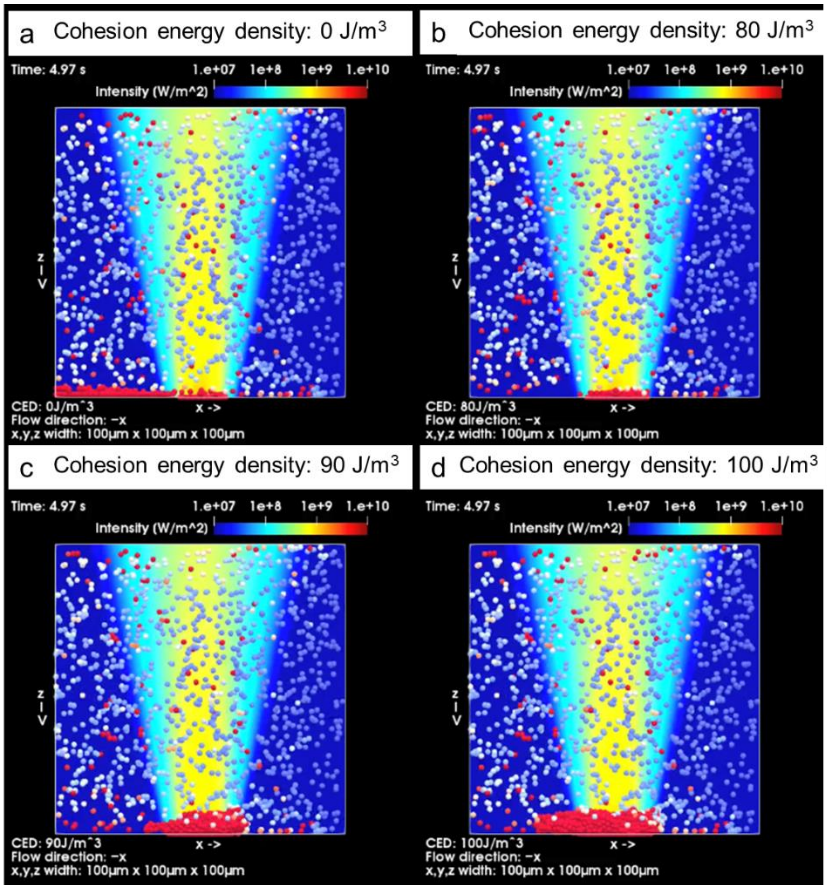

Supplementary Figure 9 | Theoretical simulations of light-induced acceleration of antigen-antibody reaction with focusing position different from Fig. 4. Calculated result of light-induced assembly of microparticles by optical pressure in microflow channel for different cohesion energy densities between antigen and antibody on each bead (volume flow rate: 0.1μL/min, Laser power: 530 mW, laser spot position: 45 μm below the bottom of the channel). In each panel, cohesion energy was assumed to be **a**: 0 J/m^3, **b**: 80 J/m^3, **c**: 90 J/m^3, **d**: 100 J/m^3. The colour of the particle indicates the time elapsed since the particle appeared in the simulation (blue: 0 seconds, white: 1 second, red: 2 seconds).

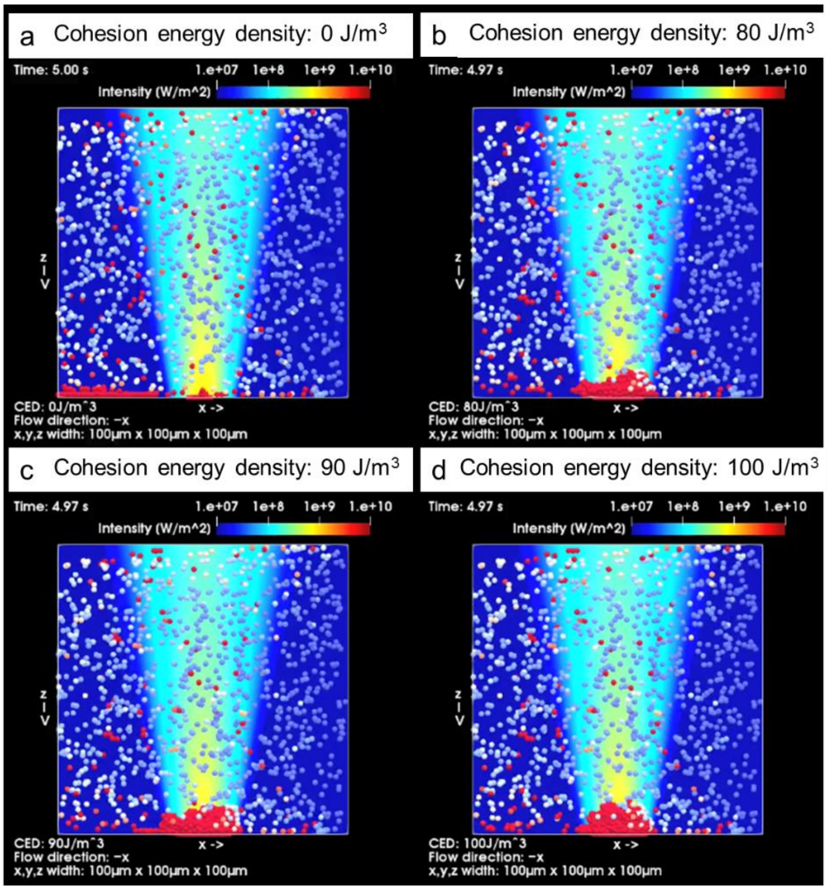

Supplementary Figure 10 | Theoretical simulations of light-induced acceleration of antigen-antibody reaction with laser power different from Supplementary Figure 9. Calculated result of light-induced assembly of microparticles by optical pressure in microflow channel for different cohesion energy densities between antigen and antibody on each bead (volume flow rate: 0.1µL/min, Laser power: 265 mW, laser spot position: 45 µm below the bottom of the channel). In each panel, cohesion energy was assumed to be **a**: 0 J/m^3, **b**: 80 J/m^3, **c**: 90 J/m^3, **d**: 100 J/m^3. The colour of the particle indicates the time elapsed since the particle appeared in the simulation (blue: 0 seconds, white: 1 second, red: 2 seconds).

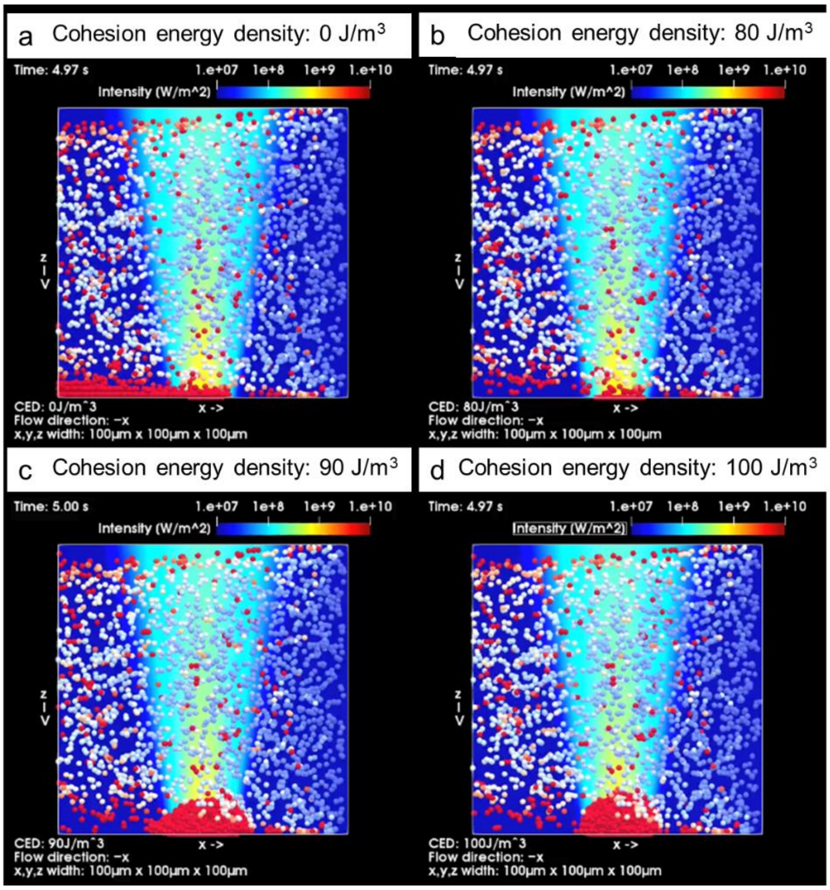

Supplementary Figure 11 | Theoretical simulations of light-induced acceleration of antigen-antibody reaction with flow rate different from Supplementary Figure 10. Calculated result of light-induced assembly of microparticles by optical pressure in microflow channel for different cohesion energy densities between antigen and antibody on each bead (volume flow rate: 0.05μL/min, Laser power: 265 mW, laser spot position: 45 μm below the bottom of the channel). In each panel, cohesion energy was assumed to be **a**: 0 J/m^3, **b**: 80 J/m^3, **c**: 90 J/m^3, **d**: 100 J/m^3. The colour of the particle indicates the time elapsed since the particle appeared in the simulation (blue: 0 seconds, white: 1 second, red: 2 seconds).
