# Supplementary material for: Attogram-level light-induced antigen-antibody binding confined in microflow
Source: Commun Biol. 2022 Oct 6;5:1053. doi: 10.1038/s42003-022-03946-0 (PMC9537419; doi:10.1038/s42003-022-03946-0)
Supplement: Supplementary file 2 — Description of Additional Supplementary Files [file 42003_2022_3946_MOESM2_ESM.pdf]

Description of Additional Supplementary Files

File name: Supplementary Data 1
Description: The source data behind the graphs in the paper.

File name: Supplementary Movie 1
Description: Video of theoretical result of light-induced assembly of microparticles by optical pressure in microflow channel, where the interparticle biding force is neglected (Cohesion energy density: 0 J/m^3 , Volume flow rate: 0.1µL/min, Laser power: 530 mW, Focal point from the bottom of microchannel (F): 65 µm). This video corresponds to Fig. 4a.

File name: Supplementary Movie 2
Description: Video of theoretical result of light-induced assembly of microparticles by optical pressure in microflow channel, where the interparticle biding force is strong (Cohesion energy density: 100 J/m^3 , Volume flow rate: 0.1µL/min, Laser power: 530 mW, Focal point from the bottom of microchannel (F): 65 µm). This video corresponds to Fig. 4d.
